# Supplementary material for: Chlorogenic Acid Isomers Isolated from Artemisia lavandulaefolia Exhibit Anti-Rosacea Effects In Vitro
Source: Biomedicines. 2022 Feb 16;10(2):463. doi: 10.3390/biomedicines10020463 (PMC8962347; doi:10.3390/biomedicines10020463)
Supplement: Supplementary file 1 [file biomedicines-10-00463-s001.zip › biomedicines-1563849-supplementary.pdf]

biomedicines

MDPI

Article

Chlorogenic Acid Isomers Isolated from *Artemisia lavandulaefolia* Exhibit Anti-rosacea Effects *in vitro*

Kyung-Baeg Roh [1], Youngsu Jang [1], Eunae Cho [1], Deokhoon Park [1], Dae-Hyuk Kweon [2], Eunsun Jung [1,*]

[1] 1Biospectrum Life Science Institute, Yongin, 16827, Korea; biosh@biospectrum.com (K.-B.R.); biogc@biospectrum.com (Y.J.); biozr@biospectrum.com (E.C.); pdh@biospectrum.com (D.P.); bioso@biospectrum.com (E.J.)
[2] Department of Integrative Biotechnology, College of Biotechnology and Bioengineering, Sungkyunkwan University, Suwon, 16419, Korea; dhkweon@skku.deu
* Correspondence: bioso@biospectrum.com; Tel.: +82-70-5117-0029

Supplementary materials

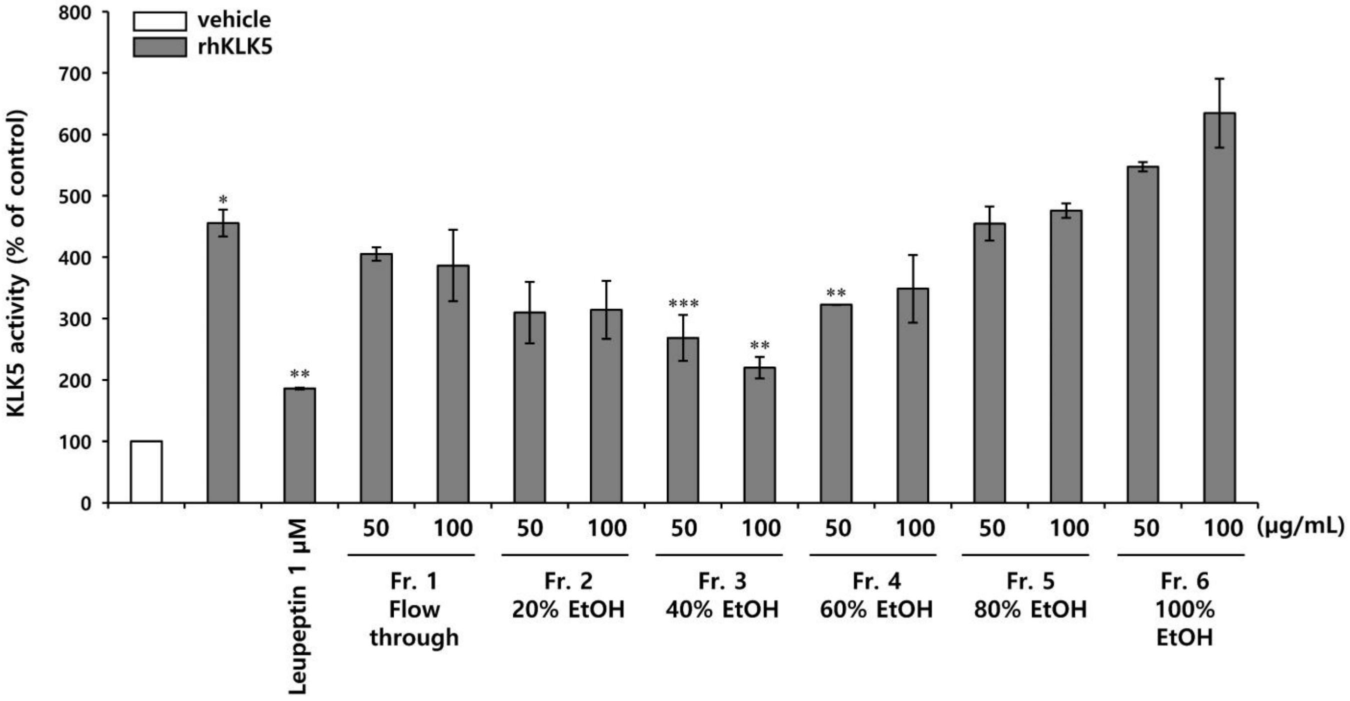

Figure S1. Assay of KLK5 inhibitory activity of fractions isolated from *A. lavandulaefolia* extract using HP-20 column chromatography. * $p < 0.01$ vs. vehicle control; ** $p < 0.01$ vs. rhKLK5-treated control; *** $p < 0.05$ vs. rhKLK5-treated control.

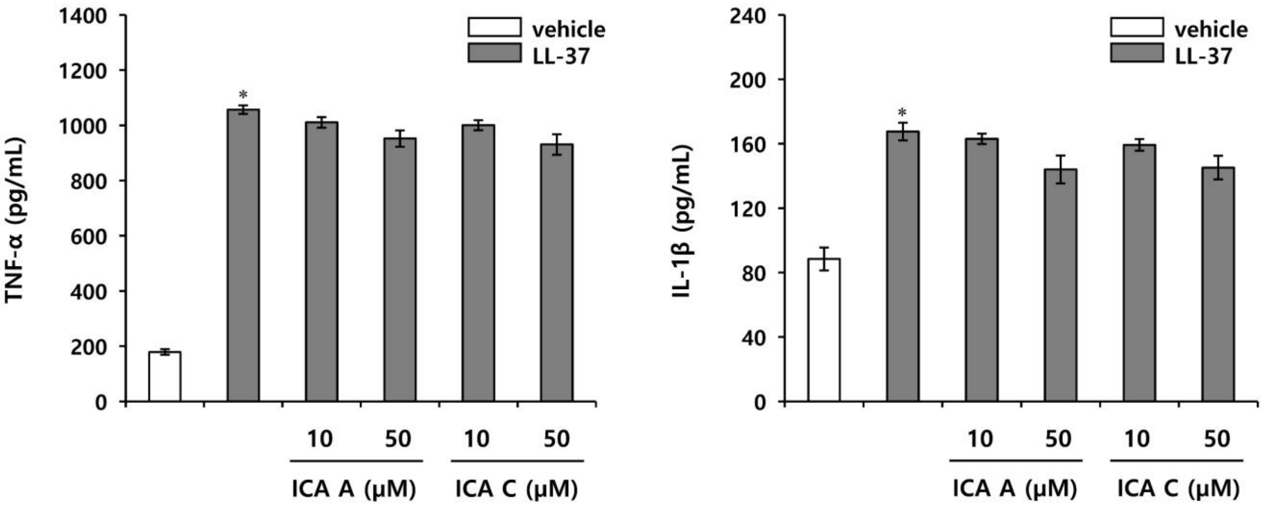

Figure S2. Effect of isolated chlorogenic acid isomers on the expression of proinflammatory cytokines induced by LL-37 in THP-1. * $p < 0.01$ vs. vehicle control.

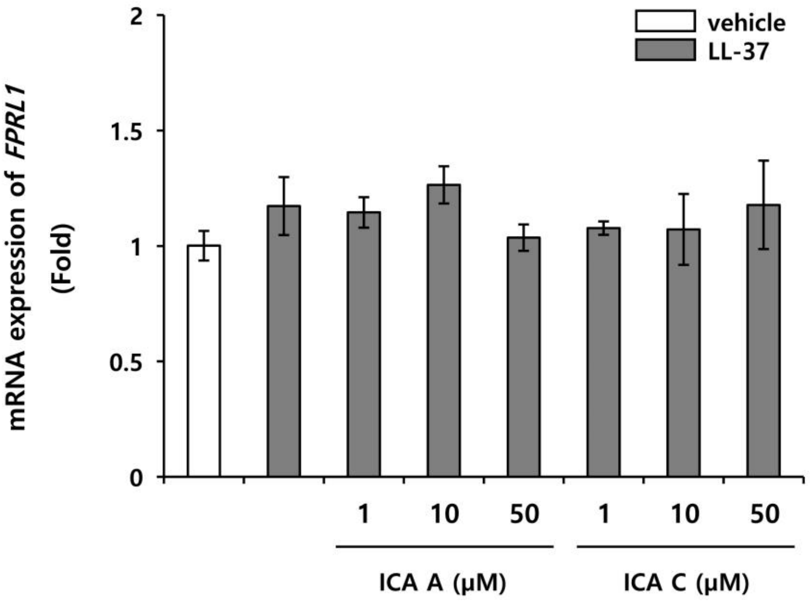

Figure S3. Effect of isolated chlorogenic acid isomers on FPRL1 mRNA expression induced by LL-37 in HMEC-1.

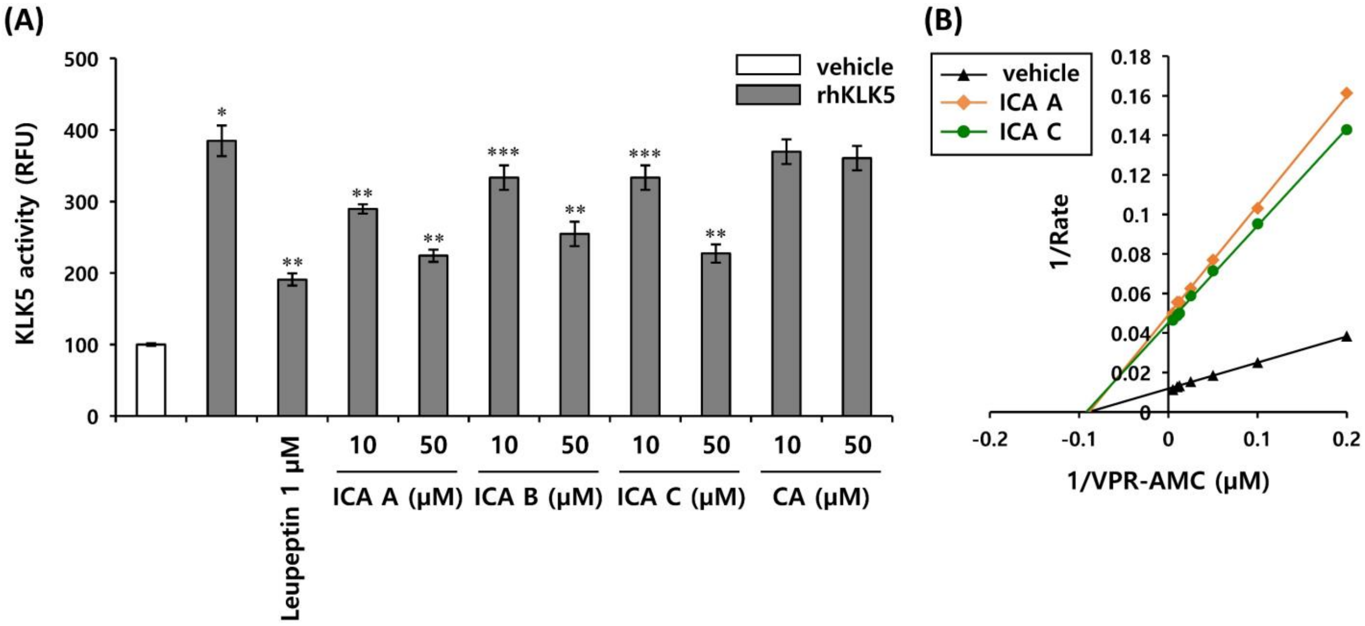

Figure S4. KLK5 inhibition activity assay. (**A**) KLK5 inhibitory activity assay of chlorogenic acid isomers and chlorogenic acid. (**B**) Lineweaver-Burk plot of isochlorogenic acids A and C against human KLK5. * $p < 0.01$ vs. vehicle control; ** $p < 0.01$ vs. rhKLK5-treated control; *** $p < 0.05$ vs. rhKLK5-treated control. ICA A, isochlorogenic acid A; ICA B, isochlorogenic acid B; ICA C, isochlorogenic acid C; CA, chlorogenic acid.

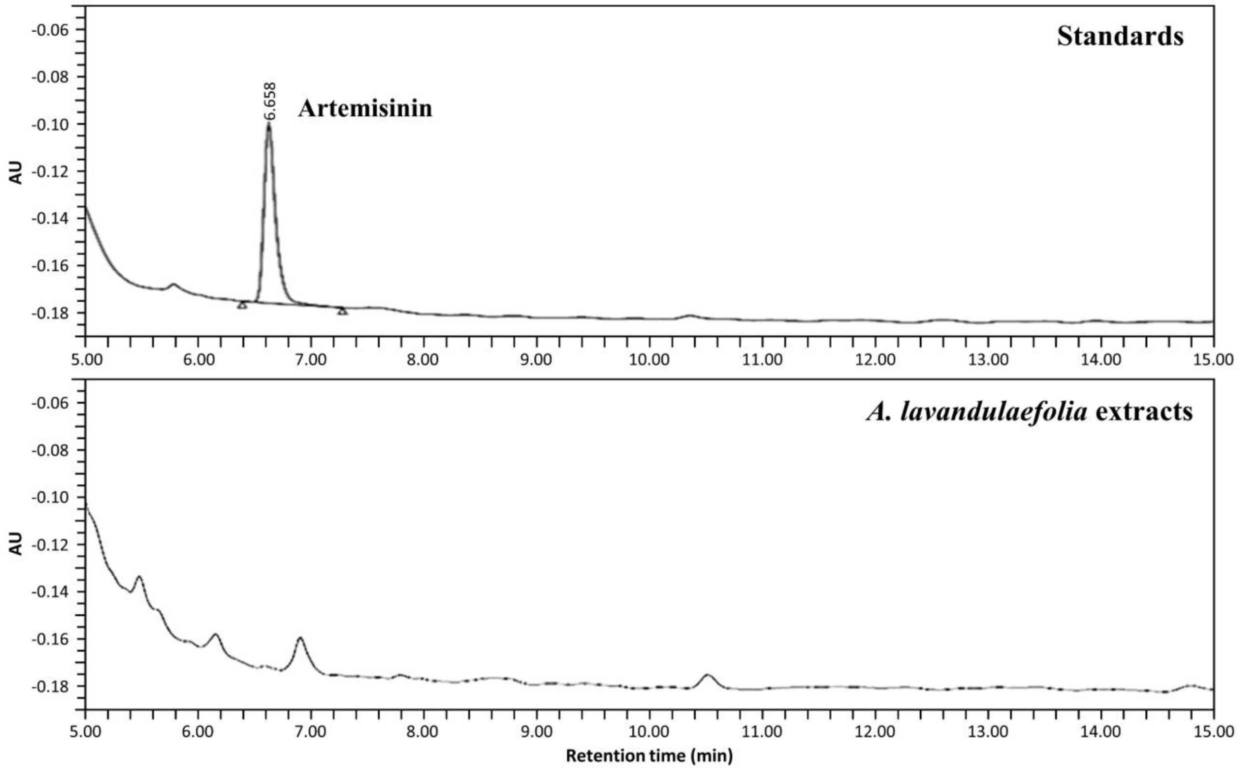

Figure S5. Analysis of artemisinin in crude *A. lavandulaefolia* extract via HPLC chromatography.
